# Deciphering the roles of tobacco MYB transcription factors in environmental stress tolerance

**DOI:** 10.3389/fpls.2022.998606

**Published:** 2022-10-24

**Authors:** Xiaoxu Li, Cun Guo, Zhiyuan Li, Guoping Wang, Jiashuo Yang, Long Chen, Zhengrong Hu, Jinghao Sun, Junping Gao, Aiguo Yang, Wenxuan Pu, Liuying Wen

**Affiliations:** ^1^ Technology Center, China Tobacco Hunan Industrial Co., Ltd., Changsha, China; ^2^ Key Laboratory for Tobacco Gene Resources, Tobacco Research Institute, Chinese Academy of Agricultural Sciences, Qingdao, China; ^3^ Kunming Branch of Yunnan Provincial Tobacco Company, Kunming, China; ^4^ Yuxizhongyan Tobacco Seed Co., Ltd., Yuxi, China; ^5^ Hunan Tobacco Research Institute, Changsha, China

**Keywords:** MYB transcription factors, tobacco, metabolism, stress tolerance, NtMYB102

## Abstract

The MYB members play important roles in development, metabolism, and stress tolerance in plants. In the current study, a total of 246 tobacco R2R3-MYB transcription factors were identified and systemically analyzed from the latest genome annotation. The newly identified tobacco members were divided into 33 subgroups together with the *Arabidopsis* members. Furthermore, 44 *NtMYB* gene pairs were identified to arise from duplication events, which might lead to the expansion of tobacco *MYB* genes. The expression patterns were revealed by transcriptomic analysis. Notably, the results from phylogenetic analysis, synthetic analysis, and expression analysis were integrated to predict the potential functions of these members. Particularly, *NtMYB102* was found to act as the homolog of *AtMYB70* and significantly induced by drought and salt treatments. The further assays revealed that NtMYB102 had transcriptional activities, and the overexpression of the encoding gene enhanced the drought and salt stress tolerance in transgenic tobacco. The results of this study may be relevant for future functional analyses of the *MYB* genes in tobacco.

## Introduction

Transcription factor is the important regulator that controls the expression of target genes, and they play important roles in plant development, metabolism, and stress response (Dubos et al., 2010; Katiyar et al., 2012; Coleto et al., 2021). The MYB transcription factor family is the one of largest transcription factor families in plants (Martin and Paz-Ares, 1997; Stracke et al., 2001). Generally, each MYB member contains a highly conserved DNA binding domain, composed of 50–52 amino acids, which binds the DNA in the form of a helix-turn-helix structure (Moyano et al., 1996). According to the repeat number of MYB domains, the MYB family members could be divided into four groups, including 4R-MYB, R1R2R3-MYB, 1R-MYB, and R2R3-MYB, and the R2R3-MYB members are the most common subgroup in plants (Stracke et al., 2001; Dubos et al., 2010). Interestingly, a conjecture is that R2R3-MYB proteins have likely evolved from R1R2R3-MYB precursors by the loss of the R1 repeat. On the contrary, the R1R2R3-MYB might evolve from R2R3-MYB by acquiring an R1 repeat. (Stracke et al., 2001; Jiang et al., 2004; Dubos et al., 2010). Since the first plant *MYB* gene was cloned from maize (Paz-Ares et al., 1987), a variety of MYB transcription factors in plants have been identified and analyzed (De Vos et al., 2006; Zou et al., 2013; Stracke et al., 2014; Qiu et al., 2019; Chen et al., 2021; Naik et al., 2021; Qin et al., 2021).

In plants, the R2R3-MYB subfamily members have been found to participate in various biological processes including plant development, secondary metabolism, and biotic/abiotic stress responses (Stracke et al., 2001; Castillejo et al., 2020; Cao et al., 2021, Ding et al., 2021). In *Arabidopsis*, 126 R2R3-MYB transcription factors have been divided into 25 subgroups, and a number of them had been reported to play an important role in primary and secondary metabolism (Allan et al., 2008; Dubos et al., 2010; Roy, 2016). In subgroup 7, as the typical MYB transcription factors, AtMYB11/PFG2, AtMYB12/PFG1, and AtMYB111/PFG3 were reported to be involved in the accumulation of specific flavonol derivatives in leaves, stems, inflorescences, siliques, and roots (Moyano et al., 1996; Stracke et al., 2007). In subgroup 6, the AtMYB75/PAP1 and AtMYB90/PAP2 transcription factors regulate the biosynthesis of anthocyanins in vegetative tissues (Quattrocchio et al., 1999; Appelhagen et al., 2011). Overexpression of *AtMYB113* or *AtMYB114* leads to a significant increase in anthocyanins production (Gonzalez et al., 2008). In addition, multiple *R2R3-MYB* genes have been reported to be involved in the synthesis of *Arabidopsis* secondary cell walls (Zhao and Dixon, 2011). In subgroup 3, AtMYB58 and AtMYB63 affect the SND1-mediated (secondary wall-associated NAC domain protein 1) transcription network regulating secondary wall formation (Zhou et al., 2009). In subgroup 16, *AtMYB83* and *AtMYB46* are both direct targets of the SND1 transcription factor and play the redundant role in the transcriptional regulatory cascade, which allows plants to form secondary walls by regulating fibers and blood vessels (Mccarthy et al., 2009).

Some *R2R3-MYB* genes have also been reported to participate in the growth and development of plants (Stracke et al., 2001; Li et al., 2020; Pucker et al., 2020). AtMYB77 of subgroup 22 was reported to control lateral root growth and development (Shin et al., 2007). In subgroup 14, AtMYB68 specifically regulates root growth, influencing the whole plant development under harsh conditions (Feng et al., 2004). AtMYB59 was reported to regulate the development of roots by regulating the cell cycle of the root tip (Mu et al., 2009). In flower development, AtMYB33 and AtMYB65, which belong to subgroup 18, facilitate both anther and pollen development, and the pollen fails to maintain vitality in *myb33/myb65* (Millar and Gubler, 2005). AtMYB120 is studied to be a pollen-specific factor, which controls the pollination of plants and the differentiation and development of pollen (Liang et al., 2013). In subgroup 21, AtMYB105/LOF2 and AtMYB117/LOF1 control the separation of the lateral stem of the plant, and AtMYB91/AS1 participates in the regulation of leaf patterning (Byrne et al., 2000; Lee et al., 2009). In subgroup 20, AtMYB2 is reported to regulate leaf senescence, and in subgroup 22, AtMYB44/MYBR1 was involved in the regulatory network of leaf senescence and ABA signaling (Guo and Gan, 2011; Jaradat et al., 2013).

Unlike animals, plants grow in a complex environment and face multiple biotic/abiotic stresses from the environment; some R2R3-MYB transcription factors have been confirmed to respond to the stresses in plants (Stracke et al., 2001; Dubos et al., 2010). For instance, AtMYB60 and AtMYB96 of subgroup 1 are reported to improve the plant drought resistance by regulating the closure of plant stomata (Seo et al., 2009; Oh et al., 2011). Similarly, in subgroup 22, AtMYB70, AtMYB73, and AtMYB74 also regulate stomatal closure to improve plants’ anti-stress ability (Jung et al., 2008). In addition, several R2R3-MYB members are also involved in cold, salinity, and wounding stresses. In subgroup 2, AtMYB2 is reported to increase plant salt resistance through ABA signaling, while AtMYB15 is involved in the regulation of cold tolerance; *myb15* increased plants’ tolerance to cold stress whereas its overexpression reduced plants’ cold tolerance (Abe et al., 2003; Agarwal et al., 2006). In defense response, AtMYB102 keeps plants from being damaged by herbivore *Pieris rapae*. AtMYB41 is reported to be involved in the negative regulation of short-term transcriptional response to osmotic pressure, and AtMYB72 is essential for *Arabidopsis* to fight against various fungal and bacterial diseases (De Vos et al., 2006; Cominelli et al., 2008; Van Der Ent et al., 2008; Segarra et al., 2009). Besides, in subgroup 22, overexpression of *AtMYB44* could promote *Botrytis* infection, and AtMYB44 positively regulates the disease resistance of *Pseudomonas syringae* in *Arabidopsis* through the salicylic acid signaling pathway (Jung et al., 2008).

Tobacco is not only a considerable economically valuable crop but also a well-studied model organism. However, extreme environment and diseases have always been the potential threat to tobacco yield and quality. Therefore, the study for systematic analysis of the MYB transcription factor family in tobacco is of great significance for the research of tobacco secondary metabolism, growth development, and resistance to biotic/abiotic stresses. With the publication of multiple plant genome sequences, a new insight combining bioinformatics and molecular biology began to identify and analyze the R2R3-MYB transcription factor family and their functions. So far, the function of the R2R3-MYB protein has been discovered and verified in a large number of plants, such as *Arabidopsis* (Dubos et al., 2010), tomato (*Solanum lycopersicum*) (Li et al., 2016), potato (*Solanum tuberosum*) (Li et al., 2019), Chinese pear (*Pyrus bretschneideri* Rehd) (Cao et al., 2016), maize (*Zea mays*) (Du et al., 2012), wheat (*Triticum aestivum*) (Wei et al., 2020), and rice (*Oryza sativa*) (Katiyar et al., 2012). However, very limited research is available on tobacco R2R3-MYB members. Here, we identified 246 *R2R3-MYB* members from the tobacco genome sequences using comparative genomic and molecular biology methods, then inferred the function of R2R3-MYB protein from phylogenetic trees, collinearity, and expression patterns. This study will provide a solid foundation for further functional studies on the function of R2R3-MYB members in tobacco growth, metabolism, and biotic/abiotic stresses.

## Materials and methods

### Identification and classification analysis of tobacco R2R3-MYB members

Version 4.5 of the genome sequence annotations of tobacco (*Nicotiana tabacum L.*) were downloaded from the SGN database (https://solgenomics.net/). The previously reported AtMYB full-length protein sequences (Stracke et al., 2001; Dubos et al., 2010) were obtained from The Arabidopsis Information Resource (TAIR, https://www.arabidopsis.org/) and used as queries to perform the BLASTP search against the annotated tobacco protein databases with an E-value cutoff of 0.01. Furthermore, with the HMM profile (PF00249), the HMM search was performed against the annotated tobacco protein databases under the E-value cutoff of 0.001. The candidate sequences from the two above-described approaches were integrated, and redundant entries were removed manually. The putative MYB protein sequences were analyzed using both Pfam (https://pfam.xfam.org/) with an E-value cutoff of 1.0 and SMART (http://smart.embl.de/) with an E-value cutoff of 1.0 to detect the presence and number of the MYB domain (Letunic et al., 2020; Mistry et al., 2021).

The full-length protein sequences of *Arabidopsis* R2R3-MYB members and newly identified tobacco R2R3-MYB members were subjected to the performance of multiple sequence alignment using MAFFT v5.3 under the default settings (Katoh and Standley, 2013). The alignment of R2R3-MYB domain was visualized using TBtools (Chen et al., 2020). Subsequently, the neighbor-joining phylogenetic analysis was conducted using MAGE X based on the alignment of full-length protein sequences with a bootstrap method of 1000 replicates, substitution with the Poisson model, and pairwise deletion (Kumar et al., 2018). The phylogenetic tree was displayed using FigTree v1.3.

### Exon–intron structural analysis and identification of conserved motifs

The genomic sequences and coding sequences of *AtMYB* and *NtMYB* genes were submitted to the Gene Structure Display Server (Hu et al., 2015) to visualize their exon–intron structure. Further, the MEME Suite 5.1.1 tool (Bailey et al., 2015) was hired to explore the conserved motifs of the AtMYB and NtMYB proteins with following parameters: maximum number of motifs: 10; the optimum width of each motif: between 6 and 100 residues.

### Chromosomal localization and duplication event analysis

According to the location information provided by the *Solanaceae* database, the Perl program was adopted to display the *NtMYB* genes’ chromosomal location. The tandem gene events were displayed on the chromosomal map according to the previous definition (Li et al., 2018). Afterward, the TBtools’ Circos program (Chen et al., 2020) was recruited to analyze the synteny relationship of the orthologous genes from tobacco and five other species (including *Arabidopsis*, tomato, potato, maize, and rice).

### Promoter analysis of tobacco *R2R3-MYB* genes

The sequences 2000 bp upstream of the *R2R3-MYB* genes in tobacco were extracted from the genome sequence database. The obtained sequences were subjected to PlantCARE platform analysis to further search for the putative *cis*-elements in their promoter regions (Lescot et al., 2002).

### Expression patterns analysis

The reported RNA-seq data of tobacco tissues (Edwards et al., 2017) were downloaded from the GEO database (accession number: GSE95717). The processed expression data of the *R2R3-MYB* genes were extracted (**Supplementary Table S1**) and transformed with log2 to normalize the raw data using TBtools (Chen et al., 2020).

The RNA-Seq data of the tobacco senescent leaf were produced in our previous work (Li and Guo, 2018) and were uploaded at the NCBI Short Read Archive (SRA) under the accession number SRP102153. The plants were grown with regular practices and topping was done 60 days after transplanting. The middle leaves were collected at nine different time points and used for transcriptomic analysis. The expression data of the *R2R3-MYB* genes were retrieved (**Supplementary Table S2**) and normalized by comparing with 5 DAT (days after topping) and log2 fold change transformation. All the normalized RNA-seq data were used to illustrate Heatmap using the pHeatmap R package under the default parameters.

### Tobacco plant preparation and stress treatments

Cultivated tobacco K326 was used to analyze the expression pattern of *NtMYB* in this study. The roots, stems, flowers, upper leaves, middle leaves, and lower leaves were collected and frozen in liquid nitrogen when cultivated tobacco K326 grew to the budding stage; the samples were stored in a refrigerator at -80°C for later use.

For the drought stress treatment assays, T3 transgenic and wild-type (K326) tobacco seeds were sown into the soil and treated after about 6 weeks as with previous conditions (Li et al., 2022). The leaves of the seedlings were detached and air-dried, then the leaf weight was recorded at 180 min.

For the salt stress treatment assays, the tobacco seedlings were germinated in a solid MS medium after disinfection, then they were transferred to a liquid MS medium and adapted for 5 days. Hereafter, some of the seedlings were transferred to a 150 mmol/l NaCl liquid MS medium or absorbent filter paper for salt and drought treatment, respectively. Samples were taken at 1, 3, and 6 h after treatment and frozen in liquid nitrogen for later use. Three biological replicates were performed for each sample.

### RNA Extraction and qTR-PCR

Total RNAs from each sample were extracted using the Ultrapure RNA Kit (cwbiotech, Beijing, China), then the first-strand complementary DNA (cDNA) was synthesized using the Evo M-MLV Mix Kit with gDNA Clean for qPCR (Accurate Biotechnology, Changsha, China). Quantitative real-time PCR (qRT-PCR) reactions were performed in a Roche LightCycler 480 Real-Time PCR instrument with SYBR^®^ Green Premix Pro Taq HS qPCR Kit (Accurate Biotechnology, Changsha, China). The tobacco ribosomal protein gene *L25* (GenBank No. L18908) was used as control (Li et al., 2021). All experimental data were obtained through three technical repetitions and three biological replicates; the relative expression level was calculated by the 2^-△△CT^ method (Livak and Schmittgen, 2001). The details of the primers are provided in **Supplementary Table S3**.

### Subcellular localization

The CDS of the *NtMYB102* gene was amplified from the cDNA of tobacco root using Phanta^®^ Max Master Mix (Vazyme, Nanjing, China) and ligated to the pEasy-Blunt vector for later use; then the sequence of the *NtMYB102* encoding gene without stop codon was inserted into the PYG57 vector, which was started by the CaMV-35S promoter and contained the GFP fragment (Sun et al., 2021). The construct was transformed into an *Agrobacterium* competent cell GV3101, and transiently expressed in the leaves of *Nicotiana benthamiana*. Simultaneously, the empty vector injected leaves as a control. Three days after the injection, the leaves were soaked in DAPI staining solution to determine the location of the nucleus, as previously reported (Li et al., 2019). Fluorescence signals were captured using a Confocal Microscope (TCS-SP8 Leica, Wetzlar, Germany).

### Transcriptional activation assay

The CDS of *NtMYB102* was amplified and inserted into the *Eco*R I site of the pBridge vector, using an Infusion HD Cloning Kit (Takara, Shiga, Japan) to be fused with a GAL4 DNA binding domain. The construct and the control vector were introduced into the yeast strain AH109 separately, followed by growing yeasts on SD/-Trp, and SD/-Trp supplemented with 5-bromo-4-chloro-3-indolyl-a-d-galactopyranoside (X-a-Gal) for 4 days at 30°C. The transcriptional activation activities were evaluated based on the growth status of different transformants.

### Tobacco transgenic plant and root length analysis

The coding sequence of the *NtMYB102* gene was amplified from the cDNA and inserted into the pCHF3 vector, which was driven by the CaMV-35S promoter, to complete the construction of the overexpression vector. The pCHF3 plasmid containing the *NtMYB102* gene was transformed using the *Agrobacterium*-mediated method (Buschmann, 2016). To obtain T1 generation homozygous lines, the seeds of serval T0 independent lines were selected on 50 mg/l kanamycin MS medium with no separation. The homozygous T1 lines and wild-type (K326) seeds were sterilized and grown in a vertical MS medium for 14 days, then transferred to an MS medium with 0 or 100 mM NaCl, respectively, to observe changes in root length. The significant difference analysis was calculated using SPSS v18.0 with the *t*-test.

## Results

### Identification of R2R3-MYB members in tobacco

To identify the R2R3-MYB members in tobacco, the BLASTP and HMMER searches were performed using the previous R2R3-MYB protein sequences from *Arabidopsis* as the query. Eventually, a total of 246 *MYB* members were obtained from tobacco genome sequences. Among them, 145 *NtMYB* genes distribute unevenly on the tobacco chromosomes, while the others map on the scaffolds. To distinguish the newly identified genes, theses *R2R3-MYB* genes were named according to physical order on the chromosome and scaffolds. The detailed information could be explored in **Supplementary Table S4**.

### Multiple sequence alignment and phylogenetic analysis

In plants, the DNA binding domain with two adjacent MYB repeats is conserved in R2R3-MYB members (Jiang et al., 2004). To explore the features of the R2R3-MYB members of tobacco, the newly identified R2R3-MYB domain performed multiple sequence alignments. As the result, the tobacco R2 and R3 MYB repeats hold the conserved amino acid residues. Notably, the tobacco R2R3-MYB domain features were found to be highly similar with *Arabidopsis* members (**Supplementary Figure 1A**). In the R2 MYB repeat, three Tryptophan (W) residues were found to be conserved, while only two Tryptophan (W) residues were found to be conserved in the tobacco R3 MYB repeat; the first Tryptophan (W) was replaced by Phenylalanine (F) or others (**Supplementary Figure 1B**).

**Figure 1:**
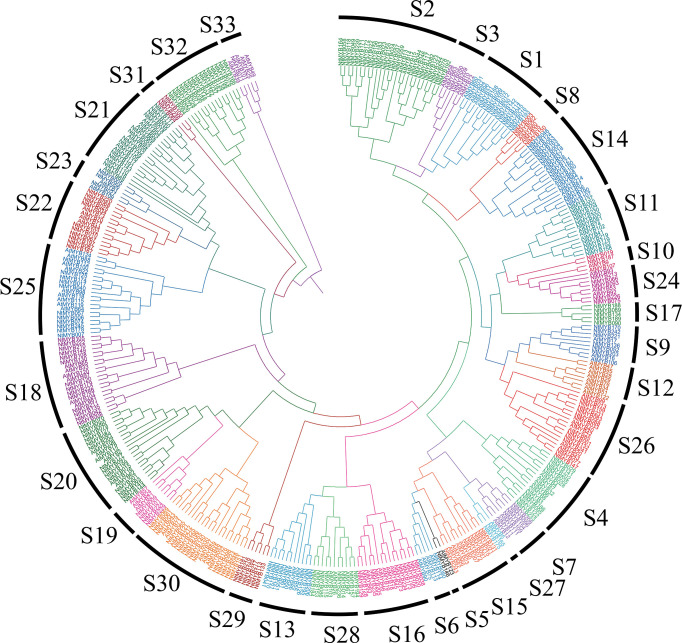
Phylogenetic analysis of tobacco R2R3-MYB family members. The tobacco R2R3-MYB members together with their *Arabidopsis* homologs were classified into 33 subgroups.

As a result, all of the R2R3-MYB members were divided into 33 subgroups, among them, the S1 to S25 subgroups were consistent with the previous reports (Dubos et al., 2010); the others were named from S26 to S33, which contained some tobacco and *Arabidopsis* R2R3-MYB members (**Figure 1**). Results showed that most of the subgroups contained R2R3-MYB members from those two species, indicating that the expansion of R2R3-MYB members may appear before the divergence of tobacco and *Arabidopsis*. Interestingly, several subgroups contained much more R2R3-MYB members from tobacco than *Arabidopsis*, such as S1, S2, and S14, implying that the duplication events might occur in those subgroups. Notably, it was found that S17, S29, S31, and S32 only harbored R2R3-MYB members from tobacco and S12 only contained members from *Arabidopsis*.

### Gene structure and conserved,motif analysis

The gene structure could provide the clues of a gene family evolution history. As a result, the intron number of studied genes was found to range from 0 to 11, and *NtMYB* genes shared the similar gene structures with *Arabidopsis MYB* genes in the same group (**Supplementary Figure S2**). Interestingly, most (82.1%) of the coding sequences of NtMYB proteins were interrupted by one or two introns, whereas 15 (6.1%) *NtMYB* genes did not hold any introns. Furthermore, more than eight introns were found in *NtMYB112*, *AtMYB88*, and *AtMYB124*, which were all clustered into S25.

In addition, the R2R3-MYB protein sequences of tobacco and *Arabidopsis* were submitted to the MEME tool to analyze the conserved motifs. As a result, a total of 10 motifs were identified, namely, motif 1–10 (**Supplementary Figures S2**, **S3**). Among them, motifs 2, 3, and 4 together constitute the R2R3-MYB domain, which could be found in all studied MYB members. Consistent with the results of gene structure analysis, the R2R3-MYB proteins in the same group usually have similar types and orders of motifs. In addition, several motifs were found to be unique to certain subgroups. For instance, motif 8 was only found in S31, while motif 9 was unique to S20, implying that these unique motifs might undertake different functions. The similarities in characteristic motifs in each group may reflect functional similarities and should be conducive to determining specific functions for each *R2R3-MYB* gene.

### Syntenic analysis of the tobacco *R2R3-MYB* gene

**Figure 2:**
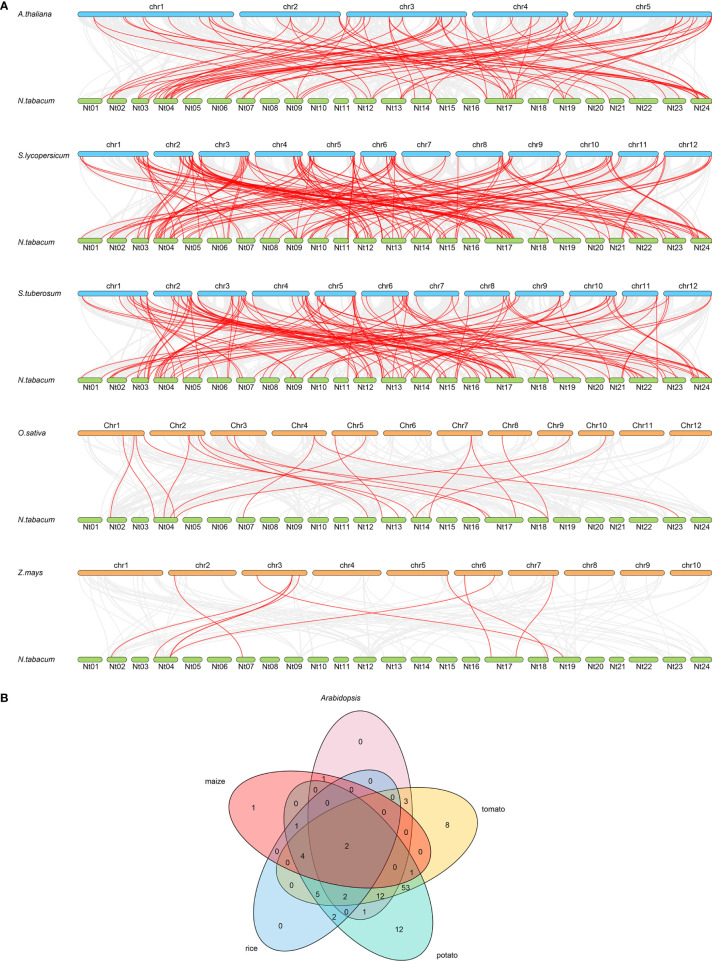
The synteny analysis of *R2R3-MYB* genes between tobacco and five representative species. **(A)** The gray line in the background represents the collinear blocks between tobacco and five representative species, while the collinear *R2R3-MYB* gene pairs are highlighted in red color. **(B)** The *R2R3-MYB* genes form the syntenic pairs between tobacco and all the other selected species, which is visualized by the Venn plot.

Syntenic analysis is important in genome sequence comparison, which reveals the genomic evolution of different species, while the syntenic pairs are predicted as orthologs and might share similar functions (Wang et al., 2021). As a result, collinearity pairs of the R2R3-MYB member were found in tobacco and five other species (**Figure 2A**). The collinear pairings between 81 of the *NtMYB* genes with MYB members in *Arabidopsis* were identified, followed by 151 *NtMYB* genes pairing with tomato, 125 *NtMYB* genes with potato, and 23 and 10 *NtMYB* genes with *MYB* genes from rice and maize, respectively. Notably, more *R2R3-MYB* collinearity pairs were found between tobacco and dicotyledonous species than monocotyledonous species. Furthermore, a total of six tobacco *MYB* genes were identified to form collinear pairs with *MYB* genes from all of the other species, indicating that these *MYB* genes may have existed before the divergence of these species (**Figure 2B**). Interestingly, 29 *R2R3-MYB* collinear pairs were predicted between tobacco and three dicotyledonous plants, but not found in the tested monocotyledonous plants, suggesting that these 29 pairs might arise after the divergence of dicotyledonous and monocotyledonous plants. The details of the tobacco *MYB* syntenic pairs can be found in **Supplementary Table S5**.

### Chromosomal distribution and duplication events

**Figure 3:**
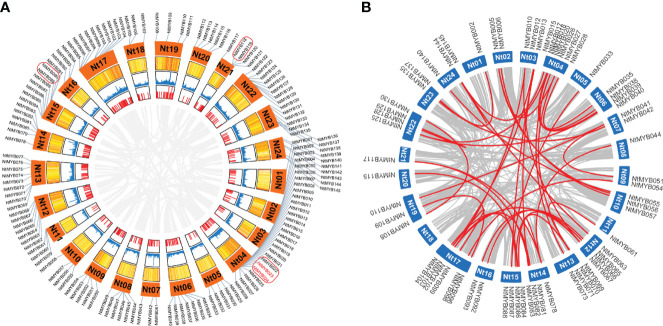
The chromosomal distribution and duplication events. **(A)** The distribution of tobacco *R2R3-MYB* genes on chromosomes. A total of 145 *R2R3-MYB* genes anchored on tobacco chromosomes. The red box suggests the gene cluster, while the tandem duplication gene pair is colored red. **(B)** The tobacco *R2R3-MYB* genes segmental duplication events and inter-chromosomal relationships. The 42 segmental duplication pairs of *NtMYB* genes are predicted by MCScanX and linked by the red lines, respectively. In addition, the gray lines stand for all putative sefmental duplication pairs in the tobacco genome sequences.

The chromosomal location information of 246 *NtMYB* genes was obtained from the SGN database and visualized using the R (**Figure 3A**). As a result, Chromosome 04 harbored the most *R2R3-MYB* genes, while Chromosomes 01, 07, 11, 16, and 18 were found to hold only three *R2R3-MYB* genes. According to the previous definition of tandem gene events (Li et al., 2018), a total of seven clusters (*NtMYB022/023*, *NtMYB023/024*, *NtMYB089/090*, *NtMYB118/119*, *NtMYB155/156*, *NtMYB171/172*, *NtMYB188/189*) were identified, the first four of which were located on chromosomes and the others were found on scaffolds. Furthermore, *NtMYB022/023* and *NtMYB171/172* were found to arise from the tandem duplication events (**Figure 3A**).

The gene segmental duplication event served as the important access for plants to acquire new genes and gene family expansion. As results, a total of 64 tobacco *R2R3-MYB* genes were identified to form 42 segmental duplication pairs (**Figure 3B** and **Supplementary Table S6**). Notably, these results suggested that about 44% of the *NtMYB* genes may be generated by duplication events, which played the major role in the expansion of the *MYB* gene family in tobacco.

### Promoter element analysis

In a previous study, the *MYB* genes had been reported to be involved in various developmental and stress responses. Considering these clues, the *cis*-element analysis was investigated to explore the probability of these *NtMYB* genes in developmental and stress responses. To study the expression regulation of the *NtMYB* genes, the promoter region of 246 *R2R3-MYB* genes was analyzed using the PlantCARE Online toolbox. Generally, it was found that a lot of *cis*-elements involved in various developmental and stress responses were present in these tobacco *R2R3-MYB* gene promoters. Furthermore, 13 *cis*-elements were selected from the PlantCARE database for visualization (**Supplementary Figure S4**). As a results, a total of 203 (82.5%) *NtMYB* gene promoters contained ABRE (abscisic acid responsive *cis*-element), suggesting that those *R2R3-MYB* genes may function in the abscisic acid signal pathway. Besides, 203 (82.5%) *NtMYB* gene promoters harbored ERE (ethylene responsive *cis*-element), while 129 (52.4%) gene promoters possessed a salicylic acid responsive *cis*-element (TCA-element). In addition, both CGTCA-motif and TGACG-motif are related to MeJA responsive, and 184 and 185 gene promoters were detected to possess these two kinds of *cis*-element, respectively. Further, a total of 98 (39.8%) gene promoters were detected to hold the CAT-box, which was related to the development of the plant meristem. Notably, the *MYB* family members were reported to be induced by stress treatments; the stress response-related *cis*-elements of tobacco *MYB* gene promoters were also analyzed consequently. The stress-responsive *cis*-elements including MBS (MYB-binding site), TC-rich repeats, HSE (heat stress-responsive element), LTR (low-temperature-responsive element), WUN-motif (wound-responsive element), and ARE (anaerobic induction element) were found to be abundant in the promoter regions of many *NtMYB* gene promoters. Interestingly, a total of 151 *NtMYB* gene promoters were predicted to hold W-box *cis*-elements, which act as the binding sites of the WRKY transcription factor, implying these *NtMYB* genes might be regulated by a certain WRKY transcription factor. Overall, the promoters of *NtMYB* genes possess abundant *cis*-elements, suggesting that the expression of these *NtMYB* genes might be regulated by multiple factors.

### Expression analysis of the *NtMYB* genes from RNA-seq

**Figure 4:**
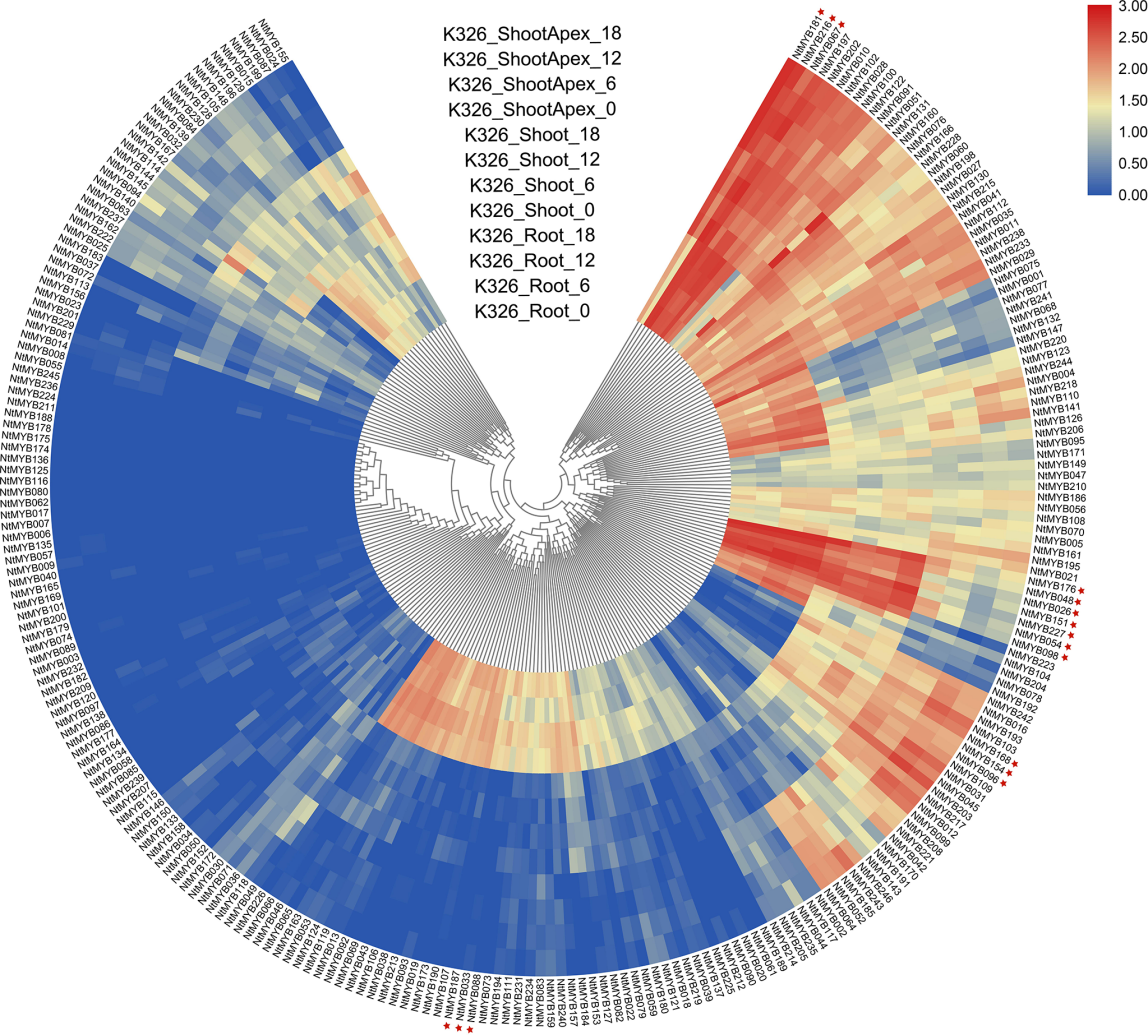
Expression profiles of the *NtMYB* genes in three different tissues (root, shoot, and shoot apex). The heatmap was constructed based on the transcriptome data of *NtMYB* genes and visualized by R.

To explore the expression pattern of the newly identified *NtMYB* genes, their RNA-seq data (GSE95717) was analyzed and visualized by R packages. As a result, the expression levels of 246 *NtMYB* genes in root, shoot, and shoot apex tissues were investigated (**Figure 4**). The results showed that several genes were expressed abundantly in the tested tissues, including *NtMYB181*, *NtMYB216*, and *NtMYB067*; while *NtMYB176*, *NtMYB048*, *NtMYB026*, *NtMYB151*, *NtMYB227*, *NtMYB054*, and *NtMYB098* were detected to be highly expressed in the shoot and root. In addition, a total of 51 genes such as *NtMYB033*, *NtMYB088*, and *NtMYB187* were only detected in the root, whereas 15 genes like *NtMYB168*, *NtMYB154*, and *NtMYB096* were found to be expressed in all tested tissues except the root. It was worth noting that 61 (24.8%) *NtMYB* genes were not detected in these tested tissues, implying those genes might function in other tissues.

**Figure 5:**
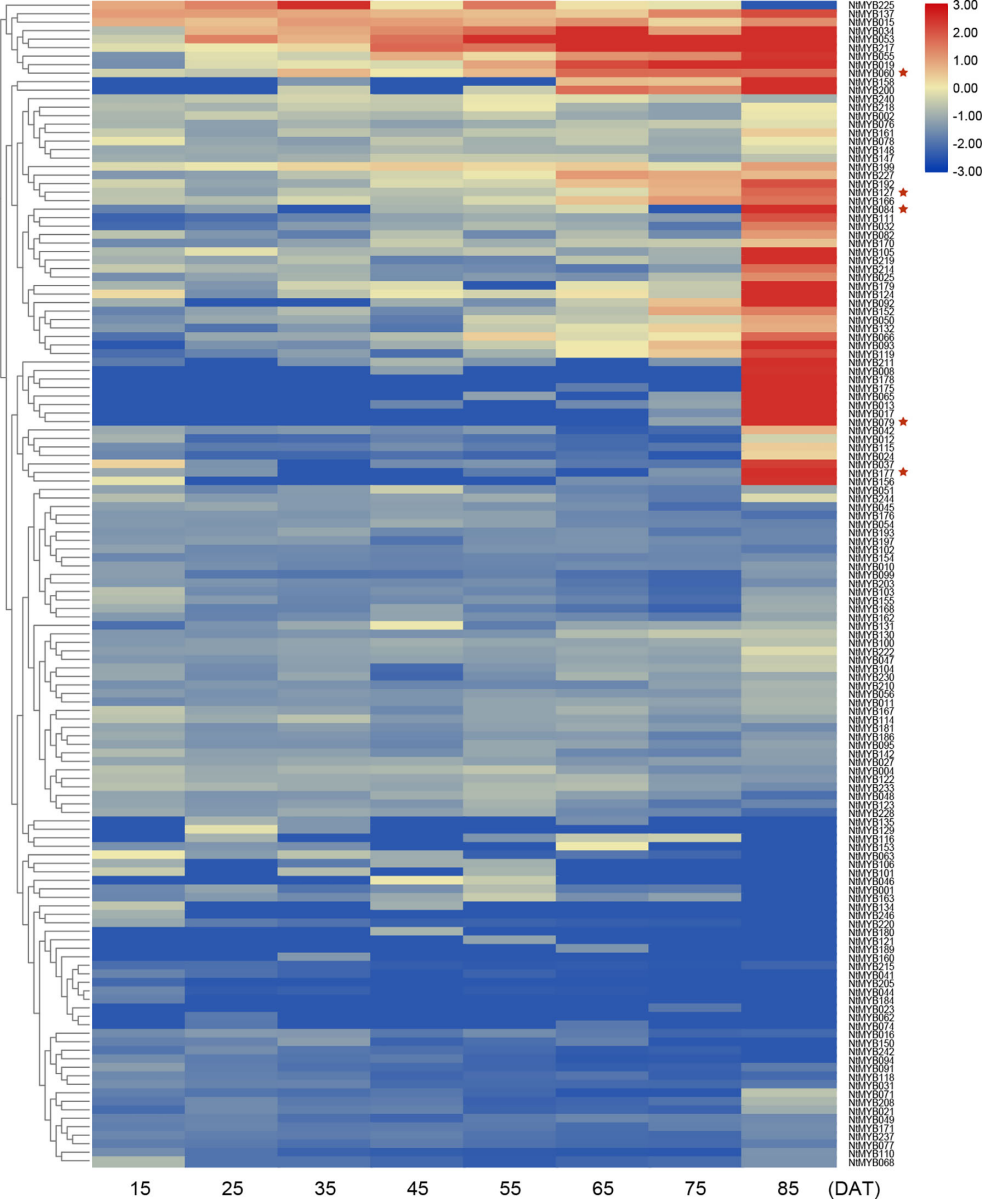
Expression profiles of the *NtMYB* genes of middle tobacco leaves in different periods. The example of middle leaf harvested from plants from 15 to 85 DAT (days after topping). The heatmap was constructed based on normalized RNA-seq data of *NtMYB* genes and visualized by R. Red indicates high expression, and blue indicates no detected expression. H indicates Honghuadajingyuan; M, middle leaves. * indicates the genes mentioned in the text.

In addition, another RNA-seq data (SRP102153) was also used to analyze the expression pattern of *NtMYB* genes in different developmental stages of tobacco leaves. As a result, a total of eight leaf growth stages were sequenced, and the expression of 57 genes increased as tobacco leaves grew (**Figure 5**). In particular, *NtMYB008* and *NtMYB178* was only expressed in the later stages of leaf growth, suggesting they might have a certain relationship with leaf senescence. *AtMYB2* was reported to contribute to the regulation of whole plant senescence (Guo and Gan, 2011), so its homologous genes were also concerned during the senescence of tobacco leaves. Combined with evolutionary analysis, it was found that genes homologous to *AtMYB2* were expressed at a high level during leaf senescence, such as the *NtMYB060*, *NtMYB079*, *NtMYB127*, *NtMYB084*, and *NtMYB177*. This finding proved the reliability of evolutionary analysis on the one hand and implied the potential function of these genes in regulating leaf senescence.

### The validation of expression patterns by qRT-PCR

**Figure 6:**
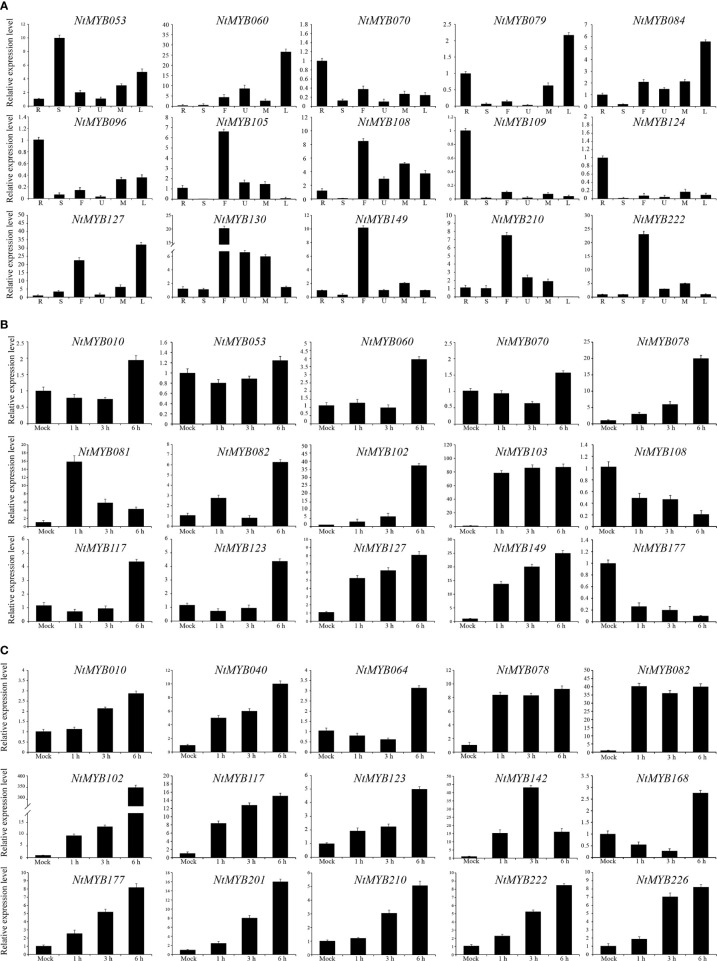
The qRT-PCR analysis of representative *NtMYB* genes. **(A)** To verify the tissue specificity expression of the representative *NtMYB* genes, the expression level of each *NtMYB* gene was calculated relative to the root. **(B)** The expression level of representative *NtMYB* genes under salt stress treatments. **(C)** The expression level of representative *NtMYB* genes under drought stress treatments.

To enrich the expression profile of the *NtMYB* genes of RNA-seq data, qRT-PCR was hired to analyze the expression changes of several representative genes. As a result, *NtMYB096*, *NtMYB109*, and *NtMYB124* were observed to highly be expressed in the root, *NtMYB053* showed abundant transcripts in the stem, and *NtMYB130* was found to be expressed globally in all tested tissues (**Figure 6A**), which were consistent with the RNA-seq data. In addition, *NtMYB060* and *NtMYB079* were detected to be upregulated during leaf senescence; on the contrary, *NtMYB210* had down-regulated expression, implying those genes might participate in leaf senescence of tobacco. Notably, qRT-PCR added the floral tissue to enrich the expression profile of tobacco *R2R3-MYB* genes; the results showed that the selected genes were expressed in varying degrees in flowers. Especially, *NtMYB105*, *NtMYB108*, and *NtMYB149* were highly expressed in flowers, suggesting they might play the key role in the flower development of tobacco.

Furthermore, a number of *R2R3-MYB* transcription factors were reported to respond to abiotic stresses in *Arabidopsis*; representative *NtMYB* genes were selected to test whether they could respond to abiotic stresses, including salt and drought stress. As a result (**Figures 6B, C**), *NtMYB078* was significantly induced by salt and drought treatments, whereas *NtMYB108* and *NtMYB177* were repressed by salt treatment. The expression of several *NtMYB* genes continued to increase under drought treatment, such as *NtMYB123*, *NtMYB201*, and *NtMYB210*. Interestingly, the expression of *NtMYB081* in response to salt stress was time-specific, reached the peak at 1 h of salt treatment, and then dropped sharply. Notably, *NtMYB102* was found to respond significantly to both drought and salt treatments, whereas *NtMYB010* was induced by drought treatment but nearly kept the original expression under salt stress.

### Subcellular localization analysis

To explore the potential function of the *NtMYB* genes, the subcellular localization of the one of the salt-responsive genes, *NtMYB102*, was analyzed (**Supplementary Figure S5**). The full-length coding sequence of *NtMYB102* without the stop codon was fused to the GFP reporter gene sequence, which was driven by the CaMV35S promoter. The *Agrobacterium* cultures with the NtMYB102-GFP fusion construct and the 35S::GFP control were transiently expressed in the leaves of *N. benthamiana*, respectively. As shown by confocal microscopy, the signal of GFP protein was found to distribute throughout the whole cell, whereas the fluorescence signal of the NtMYB102-GFP fusion protein was specifically confined within the nucleus, which was confirmed by staining with DAPI.

### The function of NtMYB102 in plant drought and salt tolerance

**Figure 7:**
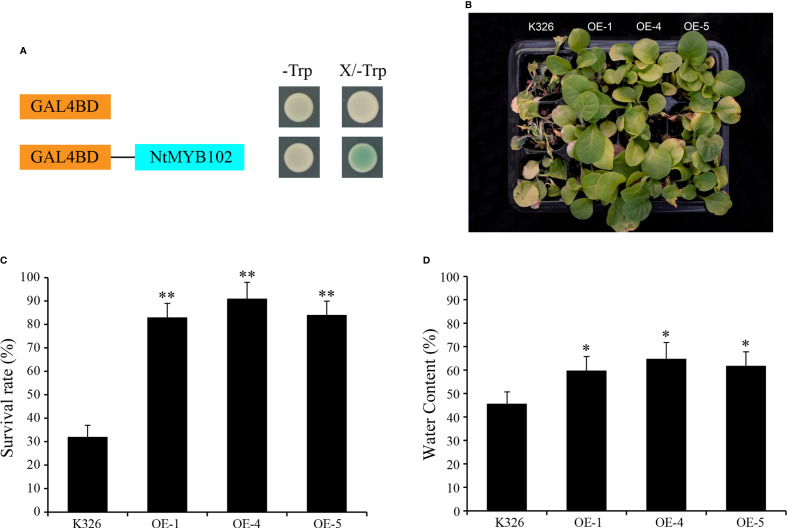
The function of *NtMYB102* in drought tolerance. **(A)** Transactivation analysis of NtMYB102 in yeast. **(B)** Phenotypes of overexpression lines and wild-type tobacco under drought stress. The overexpression lines displayed higher drought tolerance after 2 weeks of drought stress and three days of recovery. **(C)** The survival rates of the overexpression lines and wild-type tobacco. **(D)** The water content of detached leaves of overexpression lines and wild-type tobacco, which was calculated by comparing the weight of the leaves before/after the treatments. The values represent means ± SD. *p < 0.05, **p < 0.01 (*t*-tests).

As the transcriptional activation assay result, all the yeast cells grew well on a SD/-Trp medium, while on the SD/-Trp medium supplemented with X-α-Gal, the yeast cells harboring the *NtMYB102* grew well and displayed a blue color; the yeast cells containing the pBridge empty vector were not in blue color (**Figure 7A**). These results showed that NtMYB102 has transactivation activities. Furthermore, the function of *NtMYB102* gene was further examined through genetic experiments. Considering that this gene could be induced by drought and salt stresses, the overexpression lines and wild-type seedlings were firstly treated by drought stress. After the drought treatment, overexpression lines and wild-type seedlings displayed leaf wilting phenotypes, whereas the wild-type seedlings were much more extreme than those overexpression lines (**Figure 7B** and **Supplementary Figure S6**) and the survival rates of the OE-1 lines, OE-4 lines, and OE-5 lines were significantly higher than those of the wild-type seedlings (**Figure 7C**). Besides, the overexpression lines displayed higher water content than wild-type during dehydration (**Figure 7D**).

**Figure 8:**
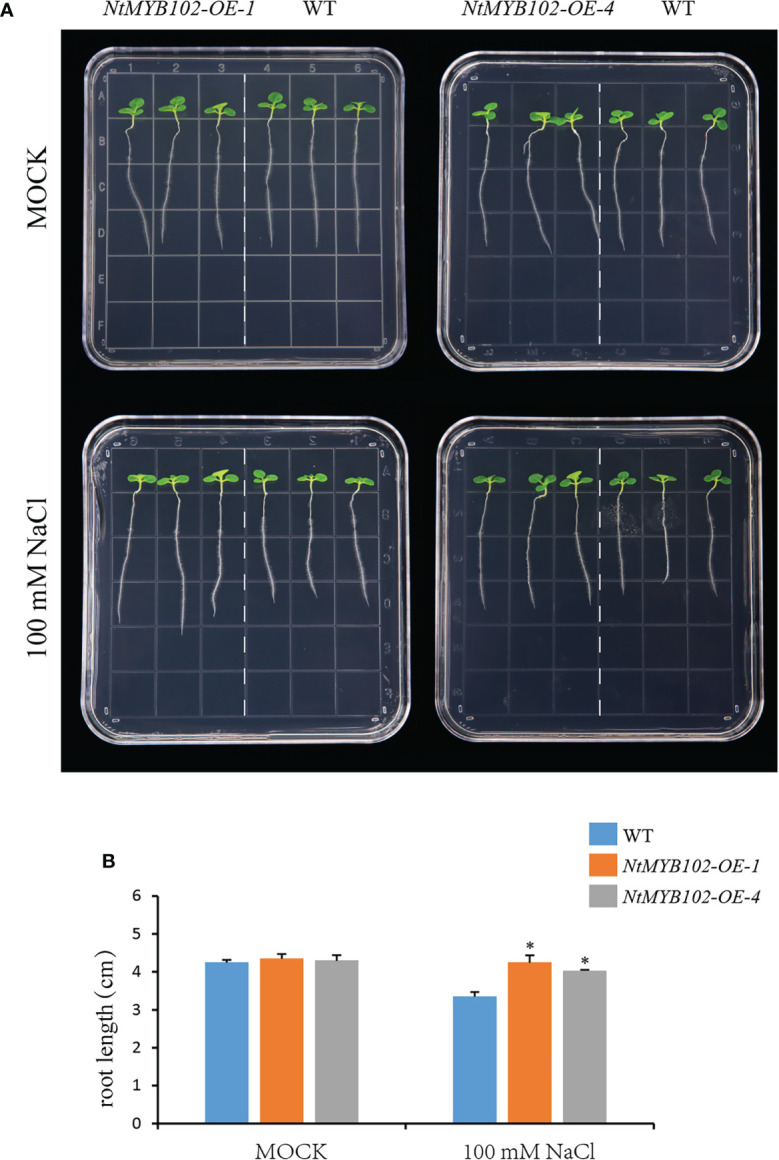
The function of *NtMYB102* in plant salt tolerance. **(A)** Root growth of wild-type and *NtMYB102* overexpression lines under 0 or 100 mM NaCl treatment. **(B)** The quantification of primary root length on medium and the data were retrieved from more than 15 plants per genotype with three biological replicates. WT, wild-type. Values represent means ± SD. *p < 0.05 (*t*-tests).

Furthermore, the salt tolerance of wild-type and *NtMYB102* overexpressing tobacco was examined *via* root elongation assay (**Figure 8**). As a result, no significant difference in root length between wild-type and the *NtMYB102* overexpressing plants was found under normal conditions. However, on 100 mM NaCl plates, longer roots of the independent overexpression lines were observed compared to the wild type. Hence, the overexpression of *NtMYB102* gene could improve the drought and salt tolerance in transgenic tobacco.

## Discussion

The MYB transcription factors play important roles in plant development, metabolism, and responding to biotic and abiotic stress (Stracke et al., 2007; Dubos et al., 2010). In this study, the newly identified R2R3-MYB members were studied through a series of analyses. In addition, the *MYB* genes homologous between *Arabidopsis* and tobacco were studied to investigate their potential functions.

A total of 246 R2R3-MYB members were identified from tobacco, and divided into 33 subgroups together with *Arabidopsis* R2R3-MYB members (**Figure 1**). The syntenic analysis could visualize the location of the homologous or orthologous genes and the presence of collinear *R2R3-MYB* genes in different species may have conserved functions, which gives an insight into the functions of the *R2R3-MYB* genes (Li et al., 2019). In the current study, we identified the collinear pairs of the *R2R3-MYB* genes in five studied species. A total of six tobacco *R2R3-MYB* genes were identified to form collinear pairs with genes from all the other species (**Figure 2**), whereas those six collinear *R2R3-MYB* genes were distributed in different subgroups (**Figure 1**), suggesting these *R2R3-MYB* genes may have existed before the divergence of these species. Gene duplication has played a very important role in the expansion of gene families (Kent et al., 2003; Cannon et al., 2004). In the current study, a total of 44 duplication events were identified in the 68 *NtMYB* genes, most (42) of which involved segmental duplication, and several (2) of which involved tandem duplication. This discovery implied that duplication events might play an important role in the evolution of the tobacco *R2R3*-*MYB* gene family.

The R2R3-MYB transcription factors have been reported to be related to plant development (Stracke et al., 2001; Pucker et al., 2020). In subgroup 14, AtMYB37, which functions in axillary meristem development (Keller et al., 2006), clustered together with NtMYB053, and their coding genes were investigated to form the collinear pair (**Figures 1**, **2**). Interestingly, *NtMYB053* was highly expressed in the stem (**Figure 4**), suggesting that it might be involved in tobacco stem development, while NtMYB124 clustered together with AtMYB68 (**Figure 1**), which specifically regulates root growth (Feng et al., 2004), and the expression profiling showed *NtMYB124* have high expression at the root (**Figure 4**), suggesting that it might be involved in the root development. In subgroup 18, AtMYB33 and AtMYB65 redundantly facilitate anther development (Millar and Gubler, 2005); NtMYB105, NtMYB222, NtMYB210, NtMYB149, NtMYB108, and NtMYB130 were clustered together with AtMYB33 and AtMYB65, and their coding genes were highly expressed in flowers (**Figures 1**, **4**), suggesting they may be involved in the development of floral organs. These results suggested functional conservation between homologous R2R3-MYB members from *Arabidopsis* and tobacco.

Besides, several MYB members have been reported to control the synthesis of the anthocyanins, proanthocyanidins, flavonols, and flavonoids in plants (Mehrtens et al., 2005; Cao et al., 2021). Notably, AtMYB11/PFG2, AtMYB12/PFG1, and AtMYB111/PFG3 from subgroup 7 were characterized as specific flavonol regulators in *Arabidopsis*; AtMYB12 controls flavonol biosynthesis mainly in the root, while AtMYB111, primarily in cotyledons (Stracke et al., 2007). Evolutionary analysis showed that NtMYB096 and NtMYB109 were clustered together with these PFG members (**Figure 1**). Meanwhile, their coding genes were predicted to form five collinear gene pairs with *AtMYB11*, *AtMYB12*, and *AtMYB111*(**Figure 2**). Further, the transcriptome and qRT-PCR data showed that *NtMYB96* and *NtMYB109* were highly expressed in roots (**Figures 4**, **6**), hinting that they might control the flavonol biosynthesis in tobacco roots. Notably, the members from subgroup 4 were identified to act as repressors of the monolignol pathway (Liu et al., 2015). NtMYB028, NtMYB029, and NtMYBB035 were found to fall into this subgroup, indicating these members may confer lignin synthesis in tobacco.

Furthermore, in subgroup 20, *AtMYB2* and *AtMYB108* were up-regulated during leaf senescence and participated in the network regulating leaf senescence (Guo and Gan, 2011; Chou et al., 2018). In this study, NtMYB060, NtMYB079, NtMYB084, NtMYB127, and NtMYB177 were found to be clustered together with AtMYB2 and AtMYB108 (**Figure 1**). The transcriptome data showed that these tobacco homologous genes were detected to be up-regulated during tobacco leaf senescence (**Figure 5**), suggesting that they might also participate in the regulation of leaf senescence of tobacco. Interestingly, although *NtMYB070* and *NtMYB084* were predicted to arise from segmental duplication events (**Figure 3B**), *NtMYB070* had lowly expressed during the senescence of the leaves (**Figure 5**), indicating these two duplicated genes might undergo subfunctionalization.

Many R2R3-MYB family members were found to confer tolerance to abiotic and biotic stresses in plants. In subgroup 1, NtMYB103 was clustered with AtMYB30, and they were detected to form a collinear gene pair (**Figures 1**, **2**). AtMYB30 had been reported to be involved in abiotic stress responses (Marino et al., 2013). Interestingly, its tobacco homologous gene, *NtMYB103*, was detected to be induced by salt treatment (**Supplementary Figure S4**), implying that NtMYB103 might be involved in abiotic stress responses of tobacco. In subgroup 2, overexpression of *AtMYB15* improves drought and salt tolerance in *Arabidopsis* (Agarwal et al., 2006). The collinearity analysis showed that *NtMYB078*, *NtMYB082*, *NtMYB117*, and *NtMYB123* in the same subgroup were investigated to be forming the collinear gene pair with *AtMYB15* respectively (**Figure 3B**). Similarly, those *NtMYB* genes were induced by multiple abiotic stress treatments (**Figures 6B, C**), implying that the R2R3-MYB members in this subgroup might also confer stress tolerance in tobacco.

In subgroup 22, NtMYB102 clustered together with AtMYB44, AtMYB70, AtMYB73, and AtMYB77 (**Figure 1**); those *Arabidopsis* members were reported to function in regulating stomatal closure and abiotic stress responses (Jung et al., 2008). Interestingly, *NtMYB102* were found to form the collinear gene pairs with those *Arabidopsis* members (**Figure 2**) and the promoter analyses revealed that the *NtMYB102* promoter region contains many ABRE *cis*-elements (**Supplementary Figure 4**), suggesting that it might be involved in ABA signalling and stress response. Notably, NtMYB102 had high transactivation activities in yeast and the NtMYB102-GFP fusion protein was in the nucleus (**Figure 7A** and **Supplementary Figure S5**). Furthermore, *NtMYB102* was significantly induced by drought and salt stresses. In addition, the overexpression analyses further demonstrated that *NtMYB102* can confer drought and salt tolerances in transgenic tobacco plant (**Figures 7**, **8**). Those clues indicated that NtMYB102 acts as a transcriptional activator to regulate gene expression in response to stresses.

## Conclusions

The systematic analysis of the tobacco genome sequences in this study was carried out to identify and characterize the *R2R3-MYB* genes; the phylogeny and expression profiling analysis implied that the tobacco *R2R3-MYB* gene family might be involved in various biological processes. The R2R3-MYB members homologous between *Arabidopsis* and tobacco were found to play conserved roles in regulating plant development and stress responses. Notably, NtMYB102 was found to be a nucleus-localized transcription factor with transactivation, and the coding gene was induced by salt treatments. Furthermore, the overexpression of *NtMYB102* in tobacco significantly enhanced the drought and salt stress tolerance of the transgenic plants.

## Data availability statement

Datasets presented in this study can be found in online repositories. The names of the repository/repositories and accession number(s) can be found in the article/**Supplementary Material**.

## Author contributions

XL, CG, and ZL conducted the research and drafted the manuscript. The other authors assisted in data collection and analysis. WP and LW conceived the research and drafted the manuscript. All authors contributed to the article and approved the submitted version.

## Funding

This research was financially supported by the China Tobacco Genome Project [110202001029(JY-12)]; The China Tobacco Hunan Industrial Co., Ltd. Technology Project (KY2022YC0010); The Key Science and Technology Program of Yunnan Provincial Tobacco Corporation (2021530000242033); The Key Science and Technology Program of Hunan Provincial Tobacco Corporation (19-23Aa01).

## Conflict of interest

XL, GW, LC, JG and WP were employed by China Tobacco Hunan Industrial Co., Ltd. CG was employed by Yunnan Provincial Tobacco Company. GW was employed by Yuxizhongyan Tobacco Seed Co., Ltd.

The remaining authors declare that the research was conducted in the absence of any commercial or financial relationships that could be construed as a potential conflict of interest.

## Publisher’s note

All claims expressed in this article are solely those of the authors and do not necessarily represent those of their affiliated organizations, or those of the publisher, the editors and the reviewers. Any product that may be evaluated in this article, or claim that may be made by its manufacturer, is not guaranteed or endorsed by the publisher.

## Supplementary material

Supplementary Sequences 1, 2 The CDS and protein sequences of all NtMYB members.

Supplementary Figure 1 The R2 and R3 MYB repeats features of MYB members from *Arabidopsis* **(A)** and tobacco **(B).** The asterisks indicate the conserved Tryptophan (W) residues in the MYB repeats.

Supplementary Figure 2 Conserved motifs and exon-intron structure organizations of NtMYB members;

Supplementary Figure 3 Detailed sequence information for each motif in NtMYB proteins;

Supplementary Figure 4 Regulatory elements in the promoter regions of tobacco R2R3-MYB genes. The color represents the number of cis-elements contained in the promoter sequence.

Supplementary Figure 5 Subcellular localization of NtMYB102. The location of the nucleus was determined by 4,6-diamidino-2-phenylindole (DAPI) staining.

Supplementary Figure 6 The expression level of *NtMYB102* gene in wild-type and two overexpression lines, the expression level of each genotype was calculated relative to the wild-type.

Supplementary Figure 7 The expression pattern of the housekeeping gene *L25* with the reported RNA-Seq data. **(A)** The expression pattern of the *L25* gene in tested tissues. **(B)** The expression pattern of the *L25* gene in tobacco middle leaves under different periods. The numbers represent the normalized data of gene expression.
