# Supplementary figures and images for: Deciphering the roles of tobacco MYB transcription factors in environmental stress tolerance
Source: Front Plant Sci. 2022 Oct 24;13:998606. doi: 10.3389/fpls.2022.998606 (PMC9638165; doi:10.3389/fpls.2022.998606)

A

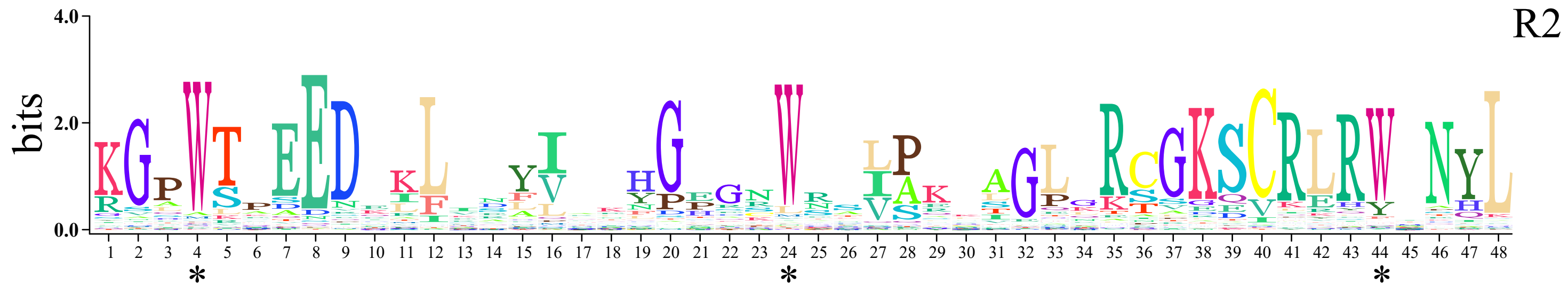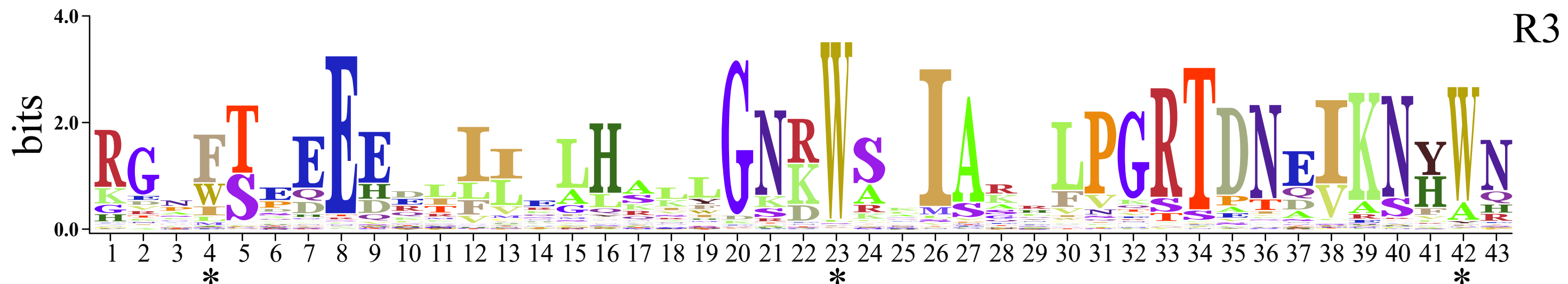

B

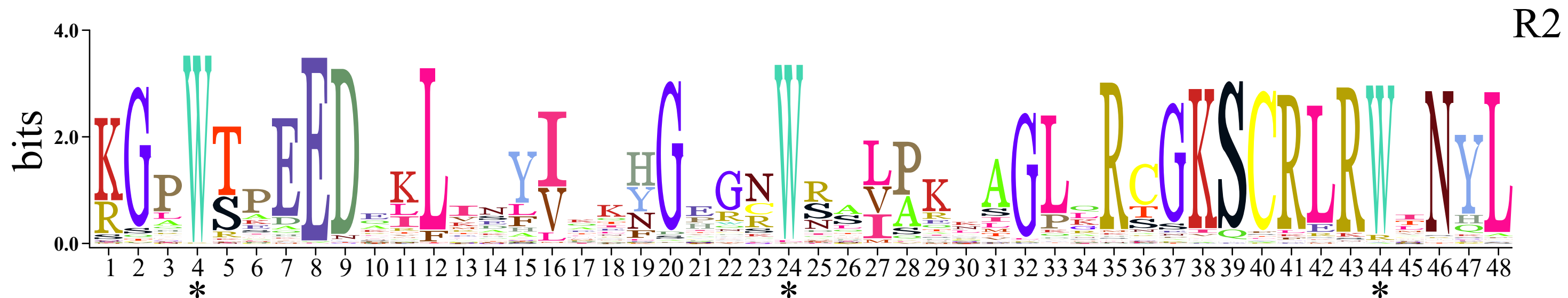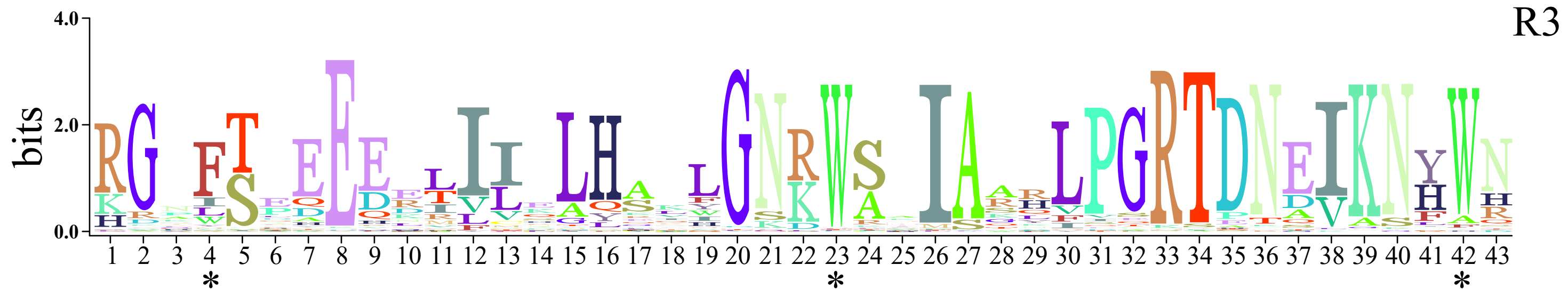

Supplement: Supplementary Sequences 1, 2 — The CDS and protein sequences of all NtMYB members. [file DataSheet_1.zip › Supplementary files/Supplementary Figure S1.pdf]

Motif 1

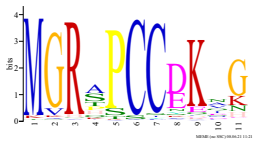

Motif 2

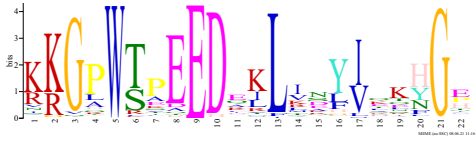

Motif 3

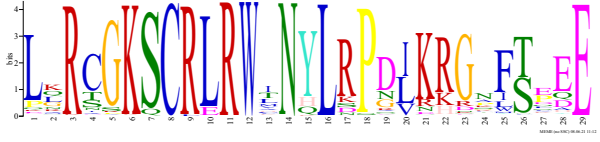

Motif 4

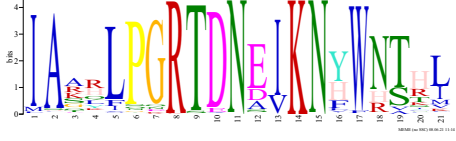

Motif 5

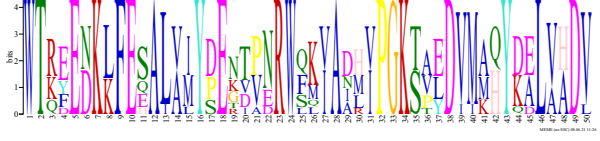

Motif 6

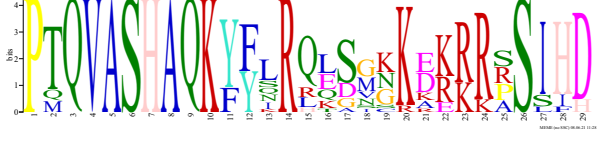

Motif 7

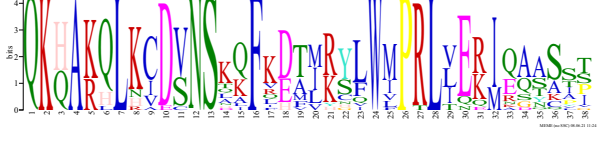

Motif 8

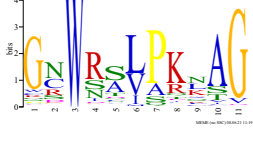

Motif 9

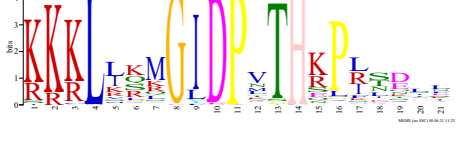

Motif 10

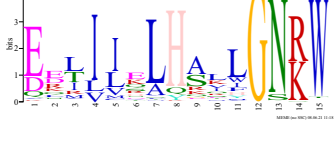

Supplement: Supplementary Sequences 1, 2 — The CDS and protein sequences of all NtMYB members. [file DataSheet_1.zip › Supplementary files/Supplementary Figure S3.pdf]

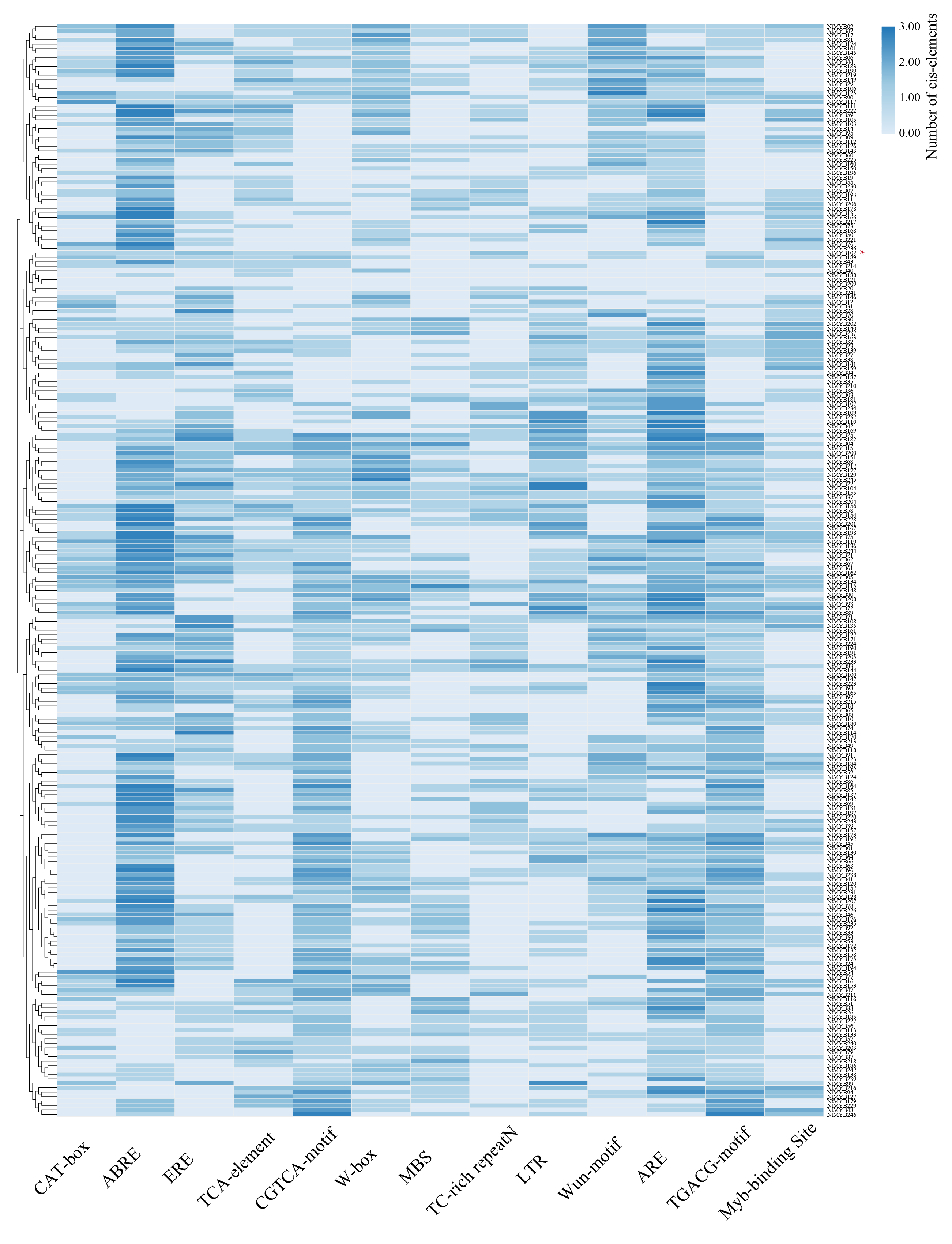

Supplement: Supplementary Sequences 1, 2 — The CDS and protein sequences of all NtMYB members. [file DataSheet_1.zip › Supplementary files/Supplementary Figure S4.TIF]

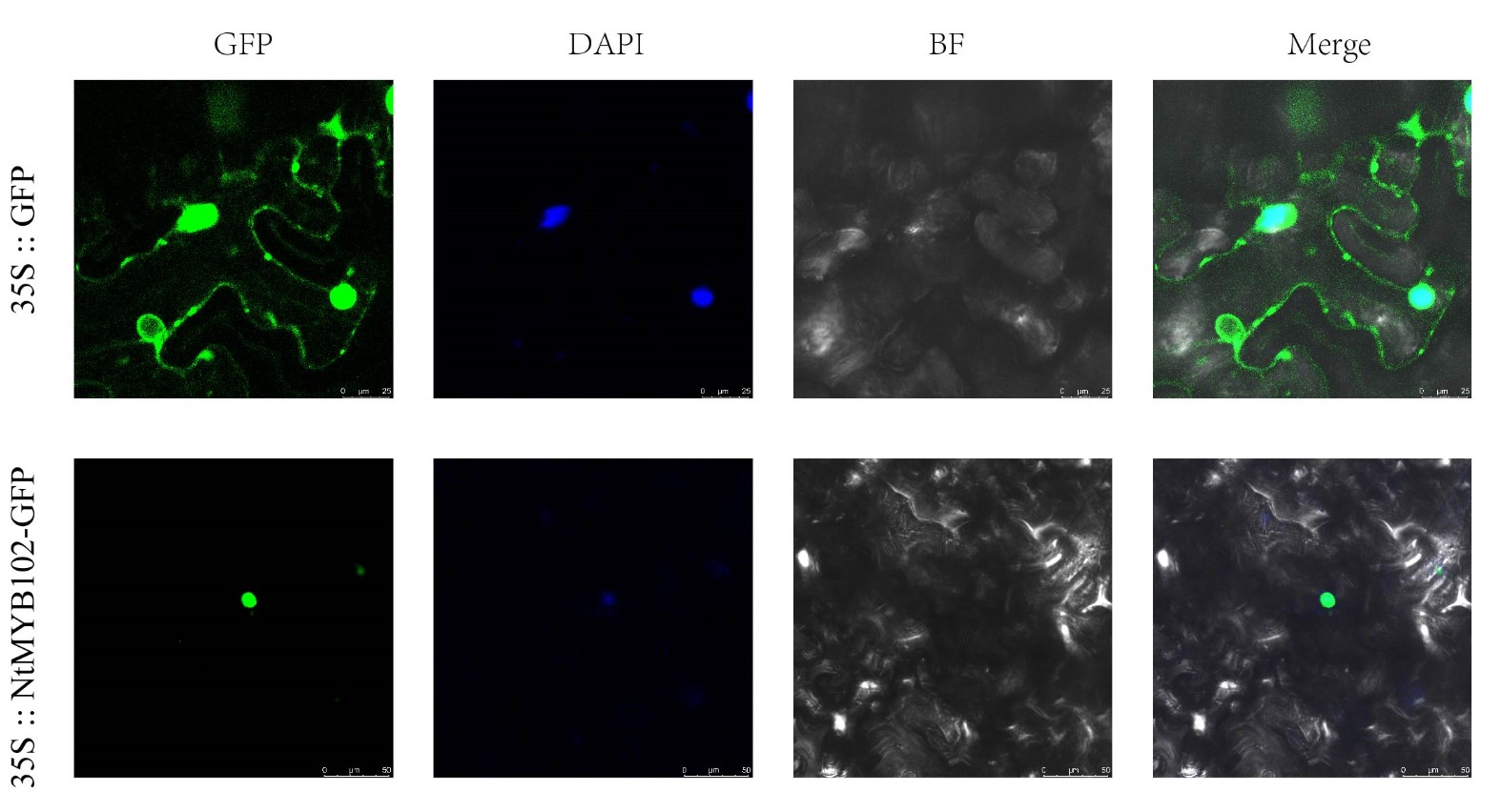

Supplement: Supplementary Sequences 1, 2 — The CDS and protein sequences of all NtMYB members. [file DataSheet_1.zip › Supplementary files/Supplementary Figure S5.jpg]

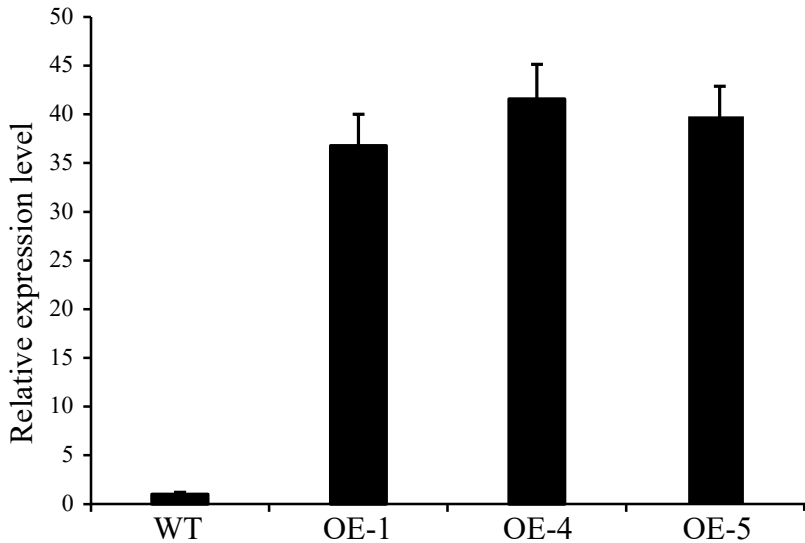

Supplement: Supplementary Sequences 1, 2 — The CDS and protein sequences of all NtMYB members. [file DataSheet_1.zip › Supplementary files/Supplementary Figure S6.pdf]

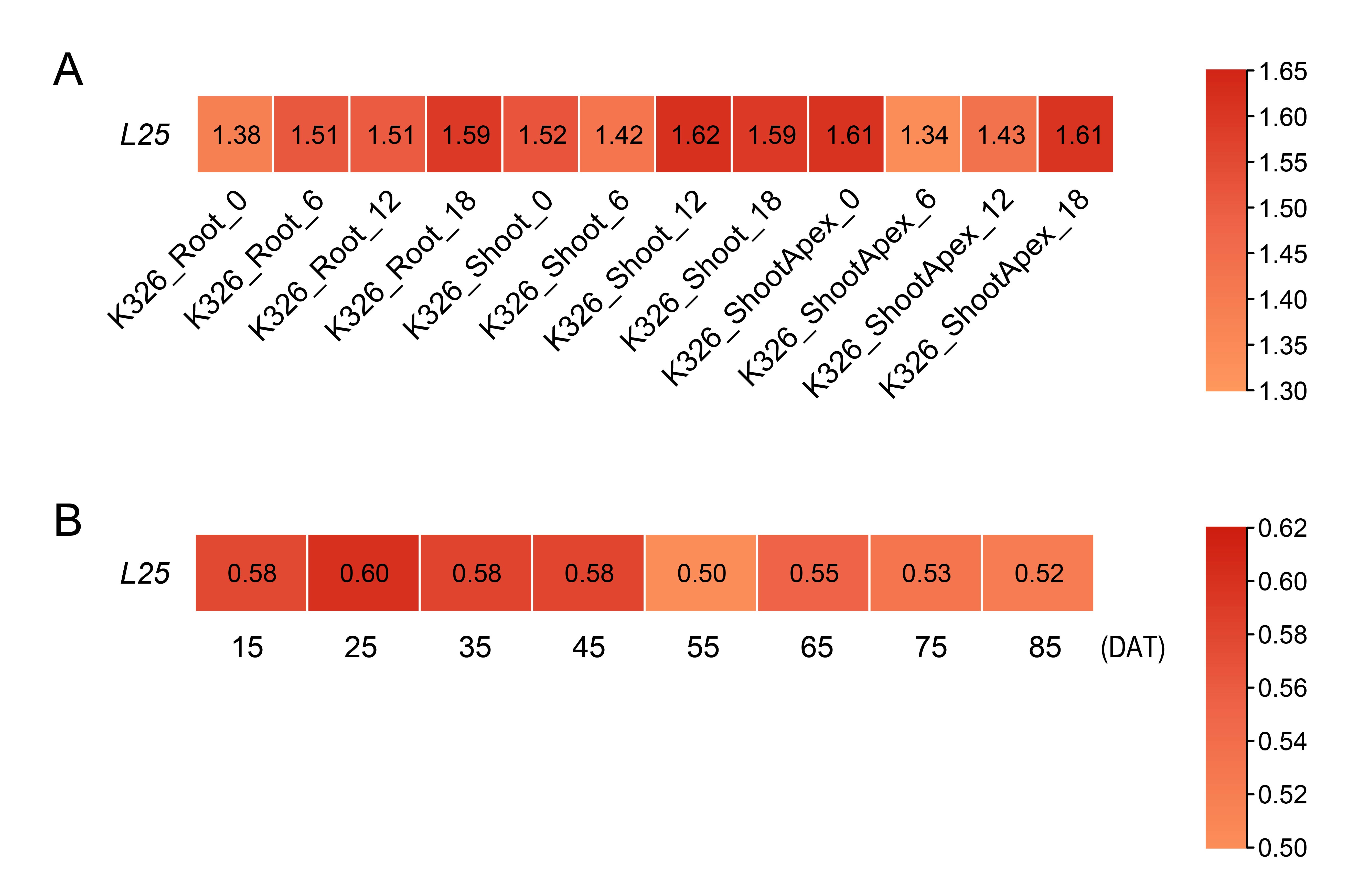

Supplement: Supplementary Sequences 1, 2 — The CDS and protein sequences of all NtMYB members. [file DataSheet_1.zip › Supplementary files/Supplementary Figure S7.jpg]
