# Supplementary material for: Deciphering the roles of tobacco MYB transcription factors in environmental stress tolerance
Source: Front Plant Sci. 2022 Oct 24;13:998606. doi: 10.3389/fpls.2022.998606 (PMC9638165; doi:10.3389/fpls.2022.998606)
Supplement: Supplementary Sequences 1, 2 — The CDS and protein sequences of all NtMYB members. [file Supplementary Figure S2.pdf › Supplementary files/Supplementary Figure S2.pdf]

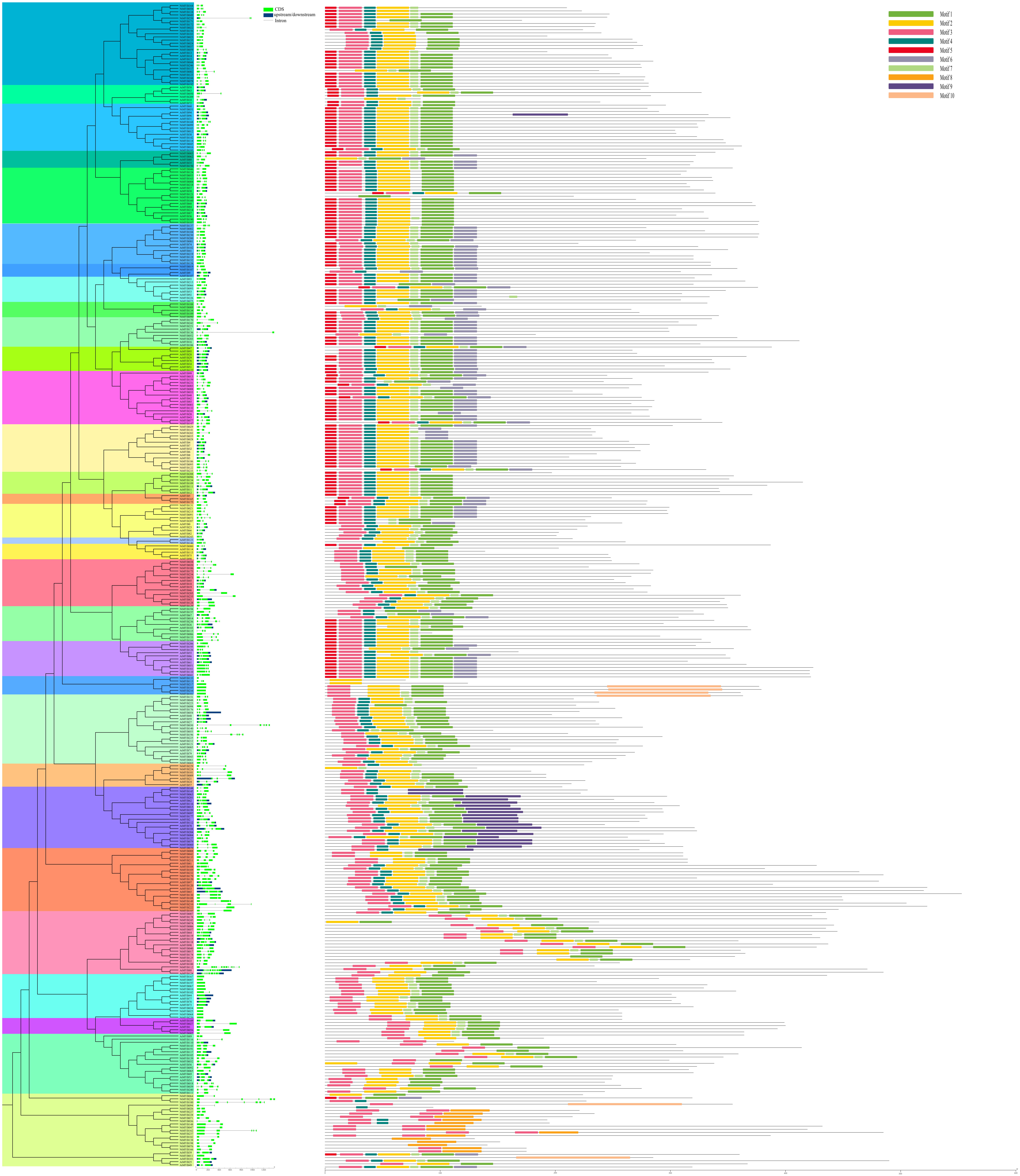
CDS
upstream/downstream
Intron
Motif 1
Motif 2
Motif 3
Motif 4
Motif 5
Motif 6
Motif 7
Motif 8
Motif 9
Motif 10
